# Cow's Milk and Rickets

**Published:** 1908-05-02

**Authors:** G. Arbour Stephens

**Affiliations:** 61 Walter Road, Swansea


(*To the Editor of* The Hospital.)

### COW'S MILK AND RICKETS.

(*To the Editor of* The Hospital.)

Sir,—I have been informed on very good authority that 90 per cent. of the milk consumed in this country is obtained from cows in calf. If this is so, is it to be wondered at that such a large number of children get rickets?

This milk in the early stages of gestation may be all right, but as the embryonic calf grows, it requires more calcium for its bones, which is obtained at the expense of the milk. Children fed on this milk are not getting the necessary elements which go to form healthy bones. This milk-drain must also have its effect on the calf, *e.g.*, by taking away such products as would help it to resist, say, tubercle.

It seems to me that an investigation into the subject is required. Farmers cannot afford to keep their calves unproductive, hence their allowing milch cows to be served six weeks after calving.

Yours truly,

G. Arbour Stephens, M.D.(Lond.).

61 Walter Road, Swansea, April 28, 1908.

